# Takotsubo Syndrome: Special Attention to Women’s Health

**DOI:** 10.21470/1678-9741-2022-0312

**Published:** 2022

**Authors:** Thiago Quinaglia Araújo Costa Silva, Maria Andréia Delbin

**Affiliations:** 1 Department of Cardiology, Laboratório de Biologia Vascular e Aterosclerose, Faculdade de Ciências Médicas, Universidade Estadual de Campinas (UNICAMP), Campinas, São Paulo, Brazil; 2 Department of Structural and Functional Biology, Laboratório de Biologia Vascular, Instituto de Biologia, Universidade Estadual de Campinas (UNICAMP), Campinas, São Paulo, Brazil. E-mail: madelbin@unicamp.br

Dear Editor,

We have read with great interest the article “Three Times Recurrent Takotsubo Syndrome: An Educational Presentation” by Silva et al.^[1]^ and we emphasize the importance of attention to women’s health. The authors presented a detailed case of a postmenopausal woman with three sparse and characteristic events of Takotsubo Syndrome (TTS) triggered by emotional stress; in two of these events, there was differential diagnosis by cineangiocoronariography. Considering the scarce documentation in the literature, the case reported may help in correlations capable of identifying similar cases, to establish more assertive and appropriate therapies during acute phase with the goal of normalizing the ventricular contractile function and preventing life-threatening complications.

It has been demonstrated that approximately 2% of all patients with suspected acute coronary syndrome (ACS) are diagnosed with TTS, the majority of whom are women over the age of 50 years. Among women only, up to 10% of patients with suspected ACS are diagnosed with TTS^[2]^. A study suggests that middle-aged and older women are being diagnosed with TTS more frequently than younger women or men of any age. Thus, the most prominent at-risk group is women aged 50 to 74 years. In addition, it has been suggested that the diagnosis rate of the condition will increase^[3]^. We highlight that the largest health threat to women over the age of 50 years is cardiovascular disease^[4]^ and that women are less likely to be diagnosed correctly, less likely to undergo the correct revascularization procedure, and less likely to survive a major cardiac event than men^[5]^.

A retrospective study carried out in Brazil showed that 85.4% of patients diagnosed with TTS were women. The morbidity and mortality rates are worthy of attention, since 41.7% of patients developed heart failure during hospitalization, the main complication of TTS. Overall, 10.4% of major adverse cardiac events were observed, of which 8.3% were in-hospital mortality^[6]^. Women appear to have risk factors that differ substantially from men. In the past years, TTS has gained attention, however the disease is still underdiagnosed. Prospective studies on TTS are largely lacking, and the condition remains incompletely understood; these facts contribute to low diagnosis awareness and its potentially severe aftermath^[7]^. Therefore, a call to health care providers on the differential diagnosis is essential, which can be optimized with a multidisciplinary approach to increase awareness. This is particularly important, as shown, in the scenario of women’s health.

## References

[1] E Silva PHR, Rosa E S J Junior, Evora PRB (2022). Three times recurrent takotsubo syndrome: an educational
presentation. Braz J Cardiovasc Surg.

[2] Akashi YJ, Nef HM, Lyon AR (2015). Epidemiology and pathophysiology of takotsubo
syndrome. Nat Rev Cardiol.

[3] Pattisapu VK, Hao H, Liu Y, Nguyen TT, Hoang A, Bairey Merz CN (2021). Sex- and age-based temporal trends in takotsubo syndrome
incidence in the United States. J Am Heart Assoc.

[4] Sorpreso ICE, Dos Santos Figueiredo FW, Ramos JLS, Zuchelo LTS, Adami F (2021). Brazilian national policy of comprehensive women's health care
and mortality during climacteric period: has anything
changed?. BMC Public Health.

[5] Roeca C, Al-Safi Z, Santoro N, Feingold KR, Anawalt B, Boyce A (2000). Endotext [Internet].

[6] Fundão NHF, Ribeiro HB, Campos CM, Seleme VB, Soeiro AM, Vieira MLC (2020). The clinical course of takotsubo syndrome diagnosed according to
the InterTAK Criteria. Int J Cardiovasc Sci.

[7] Napp LC, Bauersachs J (2020). Takotsubo syndrome: between evidence, myths, and
misunderstandings. Herz.

